# Uric acid and uric acid/creatinine ratio are associated with GDM in women undergoing IVF/ICSI

**DOI:** 10.3389/fendo.2025.1647131

**Published:** 2025-09-11

**Authors:** Yvonne Liu, Johann-Georg Hocher, Shujuan Ma, Liang Hu, Huijun Chen, Xiaoli Zhang, Fei Gong, Bernhard K. Krämer, Ge Lin, Berthold Hocher

**Affiliations:** ^1^ Fifth Department of Medicine (Nephrology/Endocrinology/Rheumatology/Pneumology), University Medical Centre Mannheim, University of Heidelberg, Mannheim, Germany; ^2^ Clinical Research Center for Reproduction and Genetics in Hunan Province, Reproductive and Genetic Hospital of CITIC-Xiangya, Changsha, China; ^3^ Medical Faculty of Charité Universitätsmedizin Berlin, Berlin, Germany; ^4^ Second Faculty of Medicine, Charles University, Prague, Czechia; ^5^ Institute of Reproductive and Stem Cell Engineering, NHC Key Laboratory of Human Stem Cell and Reproductive Engineering, Xiangya School of Basic Medical Science, Central South University, Changsha, China; ^6^ Institute of Pharmacy, Freie Universität Berlin, Berlin, Germany; ^7^ Centre for Preventive Medicine and Digital Health Baden Württemberg (CPDBW), Medical Faculty Mannheim of the University of Heidelberg, Mannheim, Germany; ^8^ European Centre for Angioscience (ECAS), Medical Faculty Mannheim of the University of Heidelberg, Mannheim, Germany; ^9^ Institute of Medical Diagnostics (IMD), Berlin, Germany

**Keywords:** uric acid, creatinine, gestational diabetes mellitus, *in vitro* fertilization, assisted reproductive technologies

## Abstract

**Introduction:**

With ongoing global lifestyle changes and economic development, the prevalence of hyperuricemia has steadily increased. Elevated levels of serum uric acid (SUA) have been linked to gestational diabetes mellitus (GDM); however, this relationship has not yet been specifically evaluated in women undergoing assisted reproductive technology (ART). Therefore, this study aimed to analyze the relationship between pre-pregnancy SUA as well as SUA to serum creatinine (SCr) ratio and GDM in women undergoing ART.

**Methods:**

This retrospective cohort study was carried out at the Reproductive and Genetic Hospital of CITIC-Xiangya in Changsha, Hunan, China, and included 1027 women who underwent their first ART treatment between 2017 and 2018. SUA levels were measured during the baseline visit prior to any ART procedures, and GDM incidence was recorded based on screening results from the oral glucose tolerance test.

**Results:**

GDM was diagnosed in 172 (16.7%) of the 1027 patients. When comparing SUA quintiles, significant differences were observed in GDM incidence, and several other parameters (including pre-pregnancy weight, BMI, blood glucose, blood pressure, SCr, lipid parameters, anti-Müllarian Hormone, follicle stimulating hormone, and testosterone). SUA was independently associated with GDM incidence after adjusting for potential confounding factors in multivariate analysis (OR 1.004, *p* = 0.003). Moreover, the SUA/SCr ratio displayed an even stronger association (OR 1.226, *p* = 0.003).

**Conclusion:**

Pre-pregnancy SUA levels – and particularly the SUA/SCr ratio – were significantly associated with GDM among women undergoing ART.

## 1 Introduction

Studies have shown that women conceiving through assisted reproductive technologies (ART), such as *in vitro* fertilization (IVF) and intracytoplasmic sperm injection (ICSI), statistically have an elevated risk for adverse pregnancy outcomes, including gestational diabetes mellitus (GDM) (1, 2). GDM is associated with many maternal and fetal complications, not only during pregnancy, but also later in life, for instance, an increased risk of type 2 diabetes mellitus and other metabolic diseases for both mother and child (3). Therefore, identifying early risk factors and enforcing early prevention especially for women at increased risk of developing GDM is of critical importance.

Hyperuricemia plays a major role in the pathogenesis of gout but has also been associated with cardiovascular and metabolic diseases, such as hypertension, chronic kidney disease (4), and diabetes mellitus (5, 6). The *American Heart Association* has defined the cardiovascular-kidney-metabolic (CKM) syndrome to describe the complex interplay between the cardiovascular, renal, and metabolic systems (7). Serum uric acid (SUA) levels can be influenced by many factors, including genetic predisposition, diets rich in fructose and/or purines (such as red meat and alcohol), overproduction in the liver, and impaired renal excretion. In China, national uric acid levels have been observed to be relatively high with an average level of 268 μmol/L in Chinese females and a rising prevalence of hyperuricemia related to lifestyle changes such as urbanization and a higher income (8). Therefore, it is very important to evaluate the possible consequences in this specific region.

The SUA/serum creatinine (SCr) ratio has also been identified as a potential marker, correlating with beta-cell function in patients with type 2 diabetes (9) and with GDM risk in women who conceived naturally (10, 11). Creatinine can indicate changes in the glomerular filtration rate, and since this ratio could be a useful parameter for the early prevention of GDM, we also analyzed its relevance in our cohort.

A systematic review and meta-analysis by Su et al., evaluating 11 studies in different countries, revealed that uric acid in the first trimester (compared to the second or third trimesters) was most strongly associated with the incidence of GDM (12). However, most previous studies measured uric acid during pregnancy, which is subject to significant changes, mainly due to the nature and difficulty of measuring parameters before conception. Our cohort of women undergoing IVF/ICSI provides a unique opportunity to evaluate pre-pregnancy values, as comprehensive baseline examinations are routinely performed prior to conception.

To the best of our knowledge, there have been no studies investigating the association between uric acid levels prior to pregnancy with GDM incidence and solely focusing on women undergoing IVF/ICSI. Therefore, the aim of this study was to investigate whether pre-pregnancy SUA levels and the SUA/SCr ratio, are independently associated with GDM in these women, potentially aiding in the early identification of women at risk and thereby an early detection of GDM.

## 2 Methods

### 2.1 Study setting

This observational study was conducted at the Reproductive and Genetic Hospital of CITIC-Xiangya in Changsha, Hunan Province, China from January 2017 to December 2018. The detailed study design has been previously published (13). The study was approved by the local ethics committee (LL-SC-2018-014) and was conducted in accordance with the Declaration of Helsinki for Medical Research involving Human Subjects. All patients provided written informed consent for participation.

### 2.2 Study participants

A total of 1027 patients were included in this retrospective study. Women who were between 18 and 40 years old were eligible if they underwent their first IVF/ICSI treatment and achieved a successful pregnancy. Patients who received oocyte donation, had uterine malformations, uterine adhesions, uterine myoma, endometriosis, untreated hydrosalpinx, Cushing syndrome, adult-onset adrenogenital syndrome, infertility caused by hypothalamic or pituitary disease, diabetes mellitus type 1 or 2 or hypertension prior to pregnancy, or missing data of pre-pregnancy SUA levels were excluded. The detailed patient selection of the initial study has been previously described (13).

### 2.3 Measurement of biochemical parameters

Patients who fulfilled the eligibility criteria received baseline tests before any fertility treatments, including a blood test. Uric acid levels were measured in all study participants at this initial visit. The biochemical analyses were routinely conducted at the hospital’s laboratory using the Beckman Coulter AU5800 automated clinical chemistry analyzer (Beckman Coulter, Brea, California, USA). SUA was determined using the uricase-peroxidase method, and SCr was measured using an enzymatic colorimetric assay. Plasma glucose was assessed using the glucose oxidase method. Standard internal quality control procedures were applied in the laboratory throughout the measurement process to ensure analytical precision. After IVF/ICSI treatment, women who achieved a successful pregnancy were followed up via telephone calls and self-reports. All participants underwent a GDM screening via a 75g oral glucose tolerance test (oGTT). GDM was diagnosed based on criteria established by *The International Association of the Diabetes and Pregnancy Study Groups* (IADPSG) (14): if either 1) fasting glucose ≥5.1mmol/L (92mg/dL), 2) one-hour glucose ≥10.0mmol/L (180mg/dL), or 3) two-hour glucose ≥8.5mmol/L (153mg/dL).

### 2.4 Statistical analysis

**Figure 1:**
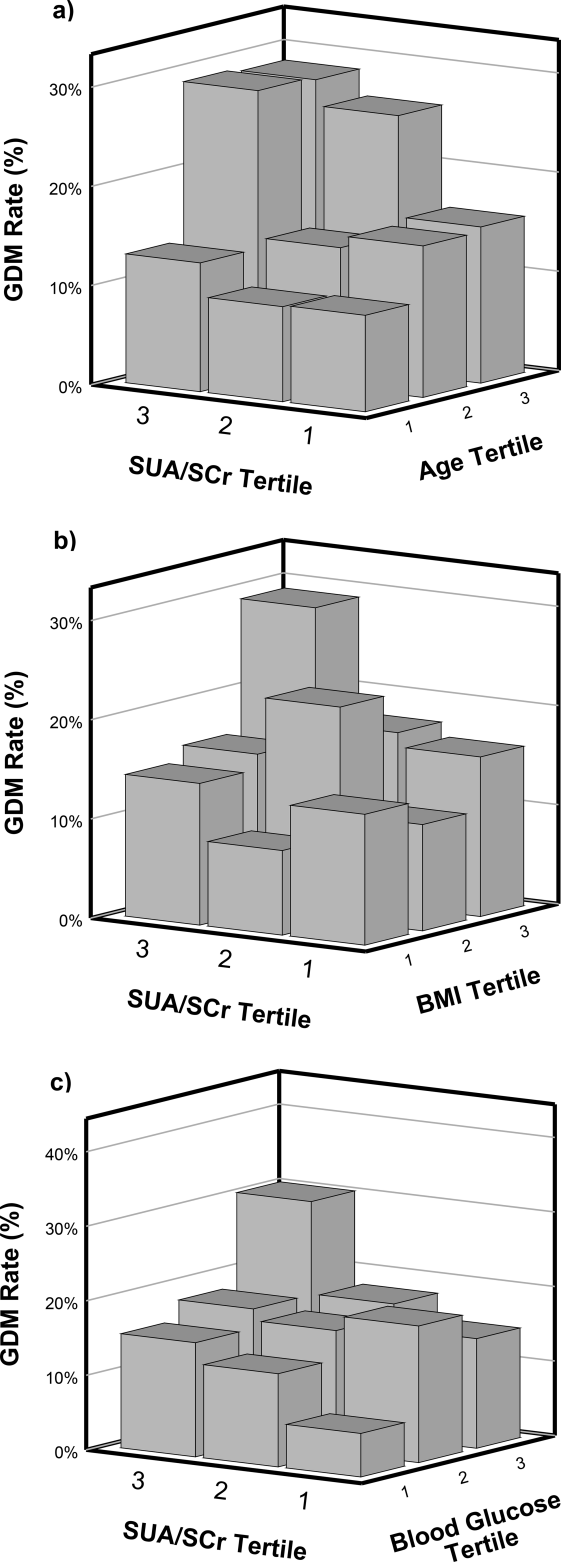
**(a–c)** 3D Plots showing interactions between GDM risk factors and SUA/creatinine ratio. BMI, body mass index; GDM, gestational diabetes mellitus; SCr, serum creatinine; SUA, serum uric acid.

**Figure 2:**
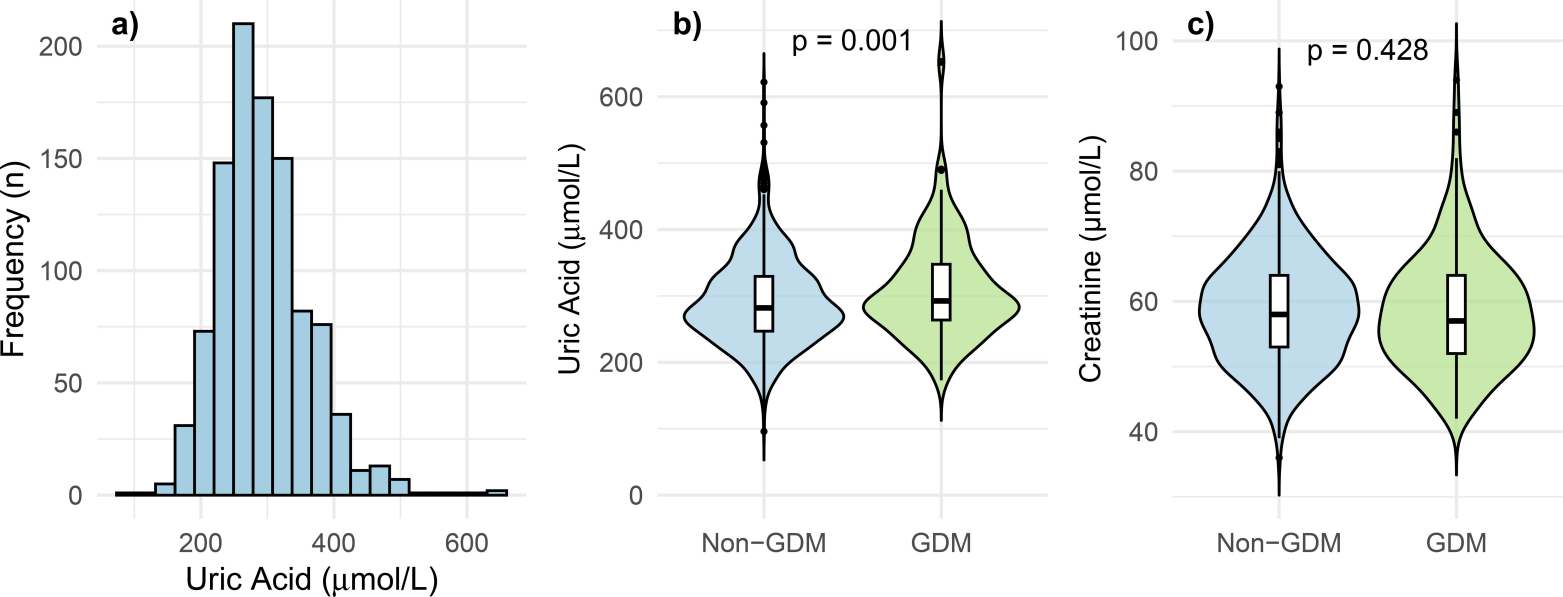
**(a, b)** Uric acid – distribution and association with GDM. **(a)** shows the non-normal distribution of uric acid in the study population in a histogram. Median (IQR): 284 (249-332) µmol/L. Violin plots **(b, c)** display summary statistics (data density distribution and median and interquartile range) of pre-pregnancy uric acid levels as well as creatinine in women who did and did not develop GDM during pregnancy. *P*-values were calculated using the Mann-Whitney U test. GDM, gestational diabetes mellitus; IQR, interquartile range.

**Figure 3:**
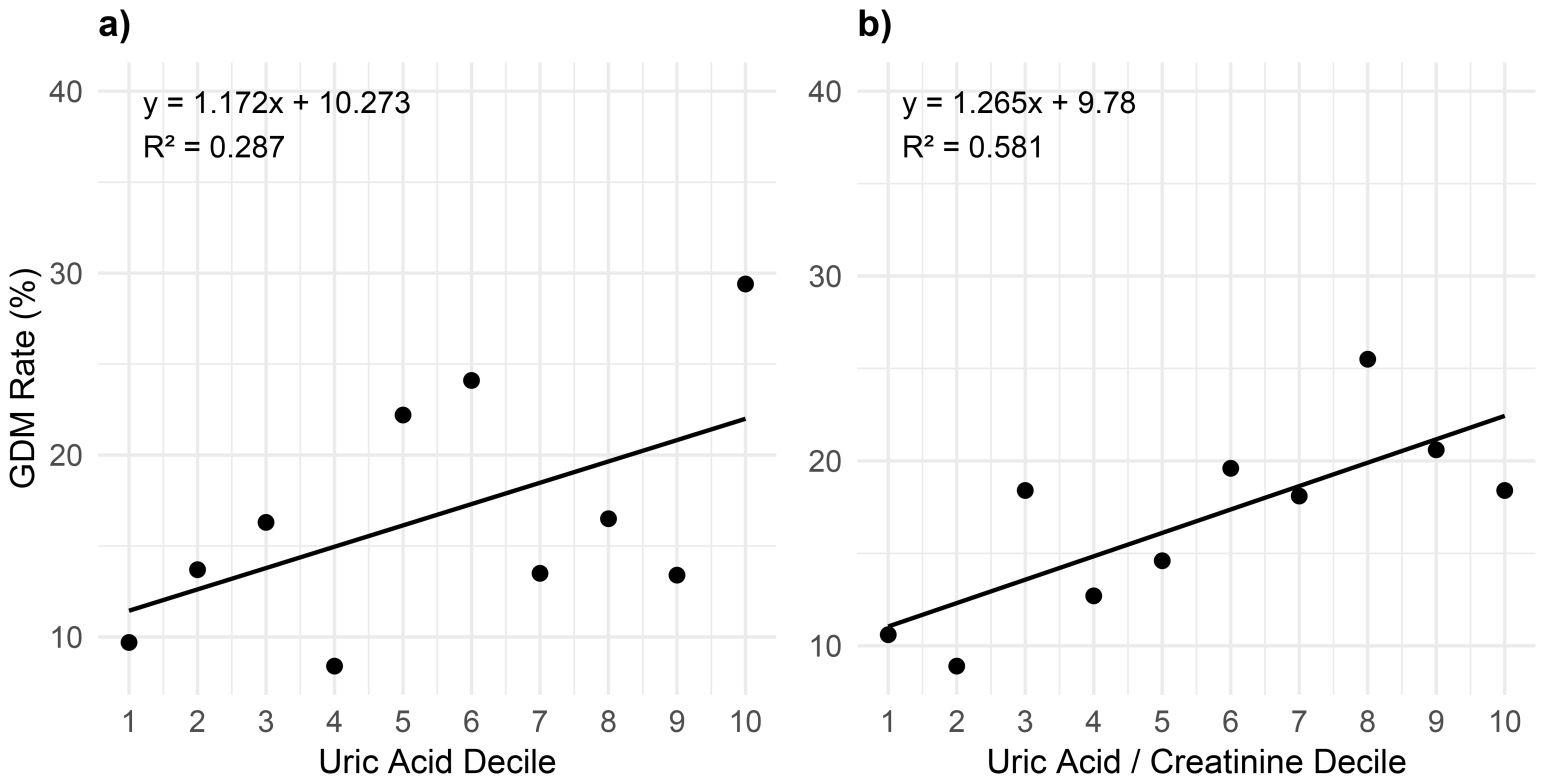
**(a, b)** Linear regression of SUA **(a)** and SUA/creatinine ratio **(b)** with GDM rate. This Figure shows the GDM rates of each concentration decile of uric acid **(a)** and of uric acid/creatinine ratio **(b)**. GDM, gestational diabetes mellitus; SUA, serum uric acid.

**Figure 4:**
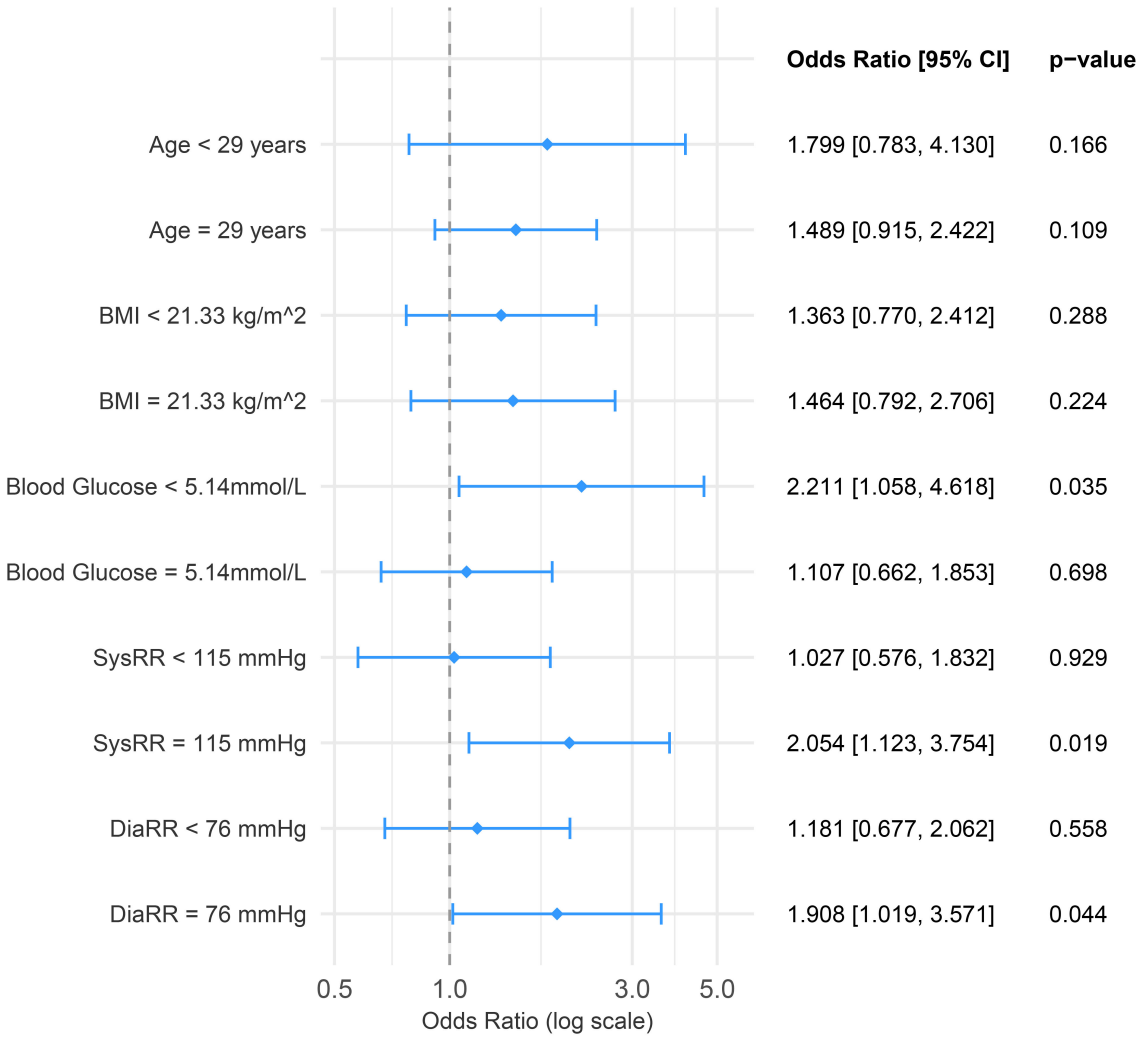
Forest plot showing subgroup analysis. Subgroups were created using the median value as a cut-off. BMI, body mass index; OR, odds ratio; SUA, serum uric acid.

Data was analyzed and figures were generated using SPSS software, version 29.0 (IBM Corporation, Armonk, New York, USA) (**Figure 1**) and R, version 4.5.0, in RStudio, version 2025.05.1 + 513 (RStudio, PBC) (**Figures 2**–**4**). *P*-values <0.05 were regarded as statistically significant. The descriptive data are shown in quintiles of uric acid in **Table 1**, and the parameters were compared using either the Chi-square (*X* ^2^) for discrete variables or the Kruskal-Wallis Test for continuous variables. We used a linear regression model to analyze the trend of GDM rate in the deciles of SUA and the SUA/SCr ratio (**Figure 3**). All results are shown with two-sided 95% confidence intervals. For multivariate logistic regression, we included known GDM risk factors (age, BMI, and pre-pregnancy blood glucose). Optimal cut-off values for SUA and the SUA/SCr ratio were calculated using receiver operating characteristic (ROC) analysis and the Youden’s index.

**Table 1:** Patient characteristics of the study population.

Parameters	All (n=1027)	Q1 (n = 204)	Q2 (n = 211)	Q3 (n = 207)	Q4 (n = 205)	Q5 (n = 199)	p-Value
Gestational Diabetes, n (%)	172 (16.7%)	24 (11.7%)	26 (12.3%)	48 (23.2%)	31 (15.1%)	43 (21.6%)	**0.002**
Age, years	29 (27–31)	29 (27–32)	29 (27-31)	29 (27-32)	29 (26-31)	29 (27-31)	0.130
Weight, kg	54.0 (49.0-59.0)	51.0 (46.5-56.9)	52.0 (28.0-55.5)	53.0 (50.0-58.0)	56.0 (51.3-60.9)	58.0 (53.0-62.5)	**<0.001**
BMI, kg/m^2^	21.48 (19.72-23.24)	20.57 (18.86-22.42)	20.83 (19.10-22.38)	21.30 (19.63-23.15)	22.03 (20.42-24.03)	22.89 (21.09-24.58)	**<0.001**
Blood Glucose, mmol/L	5.14 (4.90-5.40)	5.12 (4.89-5.39)	5.08 (4.81-5.30)	5.13 (4.89-5.42)	5.16 (4.91-5.41)	5.26 (4.97-5.51)	**0.001**
Systolic blood pressure, mmHg	115 (109-122)	115 (108-121)	114 (107-120)	115 (108-121)	116 (110-123)	117 (112-123)	**0.002**
Diastolic blood pressure, mmHg	76 (70-82)	75 (70-80)	75 (68-81)	75 (70-81)	76 (70-82)	78 (71-83)	**0.045**
Gestational hypertension, n (%)	42 (4.1%)	7 (3.4%)	6 (2.8%)	6 (2.9%)	10 (4.9%)	13 (6.5%)	0.270
Edema, n (%)	153 (14.9%)	31 (15.1%)	29 (13.7%)	32 (15.5%)	36 (17.6%)	25 (12.6%)	0.684
Anemia, n (%)	127 (12.4%)	20 (9.8%)	34 (16.1%)	20 (9.7%)	33 (16.1%)	20 (10.1%)	0.060
Polyhydramnios, n (%)	2 (0.2%)	0 (0.0%)	1 (0.5%)	0 (0.0%)	1 (0.5%)	0 (0.0%)	0.567
Oligohydramnios, n (%)	13 (1.3%)	6 (2.9%)	0 (0.0%)	3 (1.4%)	1 (0.5%)	3 (1.5%)	0.079
Placenta praevia, n (%)	7 (0.7%)	0 (0.0%)	2 (0.9%)	2 (1.0%)	2 (1.0%)	1 (0.5%)	0.693
Placental abruption, n (%)	2 (0.2%)	0 (0.0%)	0 (0.0%)	0 (0.0%)	1 (0.5%)	1 (0.5%)	0.543
Creatinine	58 (52-64)	55 (50-62)	58 (52-63)	59 (53-63)	60 (55-65)	61 (55-68)	**<0.001**
Urea nitrogen	3.80 (3.20-4.50)	3.60 (3.10-4.50)	3.80 (3.20-4.50)	3.70 (3.20-4.60)	3.90 (3.20-4.50)	3.80 (3.30-4.50)	0.278
LDL, mmol/L	2.66 (2.23-3.10)	2.53 (2.19-3.02)	2.48 (2.14-2.98)	2.64 (2.23-3.10)	2.69 (2.26-3.13)	2.93 (2.49-3.47)	**<0.001**
HDL, mmol/L	1.43 (1.22-1.66)	1.57 (1.38-1.82)	1.51 (1.33-1.70)	1.44 (1.23-1.72)	1.34 (1.15-1.58)	1.22 (1.04-1.40)	**<0.001**
Total cholesterol, mmol/L	4.13 (3.66-4.58)	4.10 (3.67-4.54)	4.00 (3.54-4.39)	4.13 (3.66-4.63)	4.09 (3.65-4.63)	4.31 (3.93-4.82)	**<0.001**
Triglyceride, mmol/L	1.00 (0.73-1.41)	0.86 (0.66-1.11)	0.84 (0.68-1.13)	0.97 (0.72-1.43)	1.09 (0.80-1.51)	1.45 (1.06-2.15)	**<0.001**
Infertility type	-	–	-	–	-	–	**0.028**
1°	602 (58.6%)	122 (59.0%)	114 (54.0%)	108 (52.2%)	128 (62.4%)	131 (65.8%)	–
2°	425 (41.4%)	84 (41.0%)	97 (46.0%)	99 (47.8%)	77 (37.6%)	68 (34.2%)	–
Fertilization method	-	–	-	–	-	–	0.936
IVF	687 (66.9%)	140 (68.3%)	136 (64.5%)	136 (65.7%)	142 (69.3%)	133 (66.8%)	–
ICSI	176 (17.1%)	37 (18.0%)	38 (18.0%)	33 (15.9%)	33 (16.1%)	35 (17.6%)	–
IVF+ICSI	164 (16.0%)	28 (13.7%)	37 (17.5%)	38 (18.4%)	30 (14.6%)	31 (15.6%)	–
Birth outcome	-	-	–	-	–		0.070
Full-term	838 (81.5%)	169 (82.4%)	164 (77.7%)	178 (86.4%)	165 (80.9%)	162 (81.8%)	–
Pre-term	174 (16.9%)	34 (16.6%)	44 (20.9%)	28 (13.6%)	36 (17.6%)	32 (16.2%)	–
Early abortion	4 (0.4%)	1 (0.5%)	3 (1.4%)	0 (0.0%)	0 (0.0%)	0 (0.0%)	–
Late abortion	7 (0.7%)	1 (0.5%)	0 (0.0%)	0 (0.0%)	2 (1.0%)	4 (2.0%)	–
Ectopic pregnancy	1 (0.1%)	0 (0.0%)	0 (0.0%)	0 (0.0%)	1 (0.5%)	0 (0.0%)	–
Delivery method	-	-	–	-	–		0.591
Normal	283 (27.6%)	58 (28.3%)	59 (28.0%)	63 (30.4%)	55 (26.8%)	48 (24.1%)	–
Cesarean	730 (71.1%)	145 (70.7%)	148 (70.1%)	144 (69.6%)	146 (71.2%)	147 (73.9%)	–
AMH, ng/mL	6.53 (4.10-10.55)	6.76 (4.29-10.60)	5.55 (3.75-8.88)	6.76 (4.07-10.22)	6.39 (4.08-11.46)	7.08 (4.44-11.79)	**0.034**
FSH, mIU/mL	5.53 (4.74-6.43)	5.66 (4.94-6.63)	5.69 (4.90-6.58)	5.50 (4.71-6.47)	5.51 (4.74-6.31)	5.34 (4.54-6.17)	**0.014**
LH, mIU/mL	3.68 (2.63-5.31)	3.65 (2.57-5.11)	3.55 (2.63-4.90)	3.76 (2.87-5.26)	3.58 (2.58-5.28)	3.87 (2.44-6.36)	0.643
Estradiol, pg/mL	33.00 (27.00-43.00)	35.00 (28.00-45.00)	34.00 (27.00-44.00)	33.00 (27.00-43.00)	32.00 (26.00-40.00)	31.00 (26.00-43.00)	0.100
Testosterone, ng/mL	0.28 (0.22-0.37)	0.27 (0.21-0.34)	0.27 (0.20-0.33)	0.30 (0.23-0.38)	0.29 (0.22-37)	0.32 (0.24-0.41)	**<0.001**
AFC	16 (12-22)	15 (12-21)	15 (12-20)	16 (13-23)	15 (12-22)	17 (13-24)	0.070

The data is represented as frequency, n (%) for categorical variables or median (interquartile range, IQR) for continuous variables. P-values were calculated using the Chi-square (*X* ^2^) test for categorical variables and Kruskal-Wallis test for continuous variables.

Quartiles of uric acid (/µmol/L): Q1 (≤239), Q2 (>239-≤272), Q3 (>272-≤302), Q4 (>302-≤347), Q5 (>347). Bold p-values indicate that the association is statistical significant (p<0.05).

AFC, antral follicle count; AMH, anti-Müllarian hormone; BMI, body mass index; FSH, follicle-stimulating hormone; GDM, gestational diabetes mellitus; HDL, high density lipoprotein; IVF, in vitro fertilization; ICSI, intracytoplasmic sperm injection; LDL, low density lipoprotein; LH, luteinizing hormone; RR, blood pressure.

## 3 Results

Of the 1027 patients included in the study, 172 (16.7%) developed GDM during pregnancy. The median maternal age was 29 years (interquartile range [IQR]: 27-31). The cohort had a median pre-pregnancy weight of 54 kg (IQR: 49–59 kg) and a median BMI of 21.48 kg/m^2^ (IQR: 19.72-23.24 kg/m^2^). Most patients (66.9%) underwent IVF treatment without ICSI, while 17.1% underwent ICSI and 16.0% underwent a combination of IVF and ICSI. A total of 730 (71.1%) patients delivered via cesarean section. Detailed patient characteristics are presented in **Table 1**. The median baseline SUA level, measured prior to any fertility treatments was 284 µmol/L (IQR: 249-332 µmol/L).

SUA levels were not normally distributed, as shown graphically in **Figure 2a** and confirmed by both the Kolmogorov-Smirnov and Shapiro-Wilk tests for normality (both *p* < 0.001). When stratifying patients by SUA quintiles, the incidence of GDM differed significantly (*p* = 0.002). Other factors that were statistically significant across SUA quintiles were weight (*p* < 0.001), BMI (*p* < 0.001), pre-pregnancy blood glucose (*p* = 0.001), systolic and diastolic blood pressure (*p* = 0.002 and *p* = 0.045, respectively), SCr (*p* < 0.001), the lipid parameters (LDL, HDL, total cholesterol, and triglycerides, all < 0.001), anti-Müllarian hormone (AMH) (*p* = 0.034), follicle stimulating hormone (FSH) (*p* = 0.014), and testosterone (*p* < 0.001) (**Table 1**).

**Figure 2b** displays a comparison of SUA levels between patients who developed GDM and those who did not (*p* = 0.001). The SUA/SCr ratio, a commonly used clinical parameter, was also significantly different between these two groups (*p* < 0.001), while SCr alone was not significantly different (*p* = 0.428) (**Figure 2c**). To explore these associations further, we conducted linear regression analyses (**Figures 3a, b**), which show a rising trend in GDM rate across SUA deciles (**Figure 3a**, r^2^ = 0.287) and an even stronger association with the SUA/SCr ratio (**Figure 3b**, r^2^ = 0.581).

Multivariate logistic regression, adjusting for known GDM risk factors (age, BMI, and pre-pregnancy blood glucose), revealed that both SUA and the SUA/SCr ratio were significantly associated with GDM (SUA: OR 1.004, *p* = 0.003; SUA/SCr: OR 1.226, *p* = 0.003) (**Table 2**). Using ROC analysis, we identified optimal cut-off values: 270.05µmol/l for SUA and 5.02 for SUA/SCr. When we dichotomized these variables and re-ran the regression, both binary indicators showed even stronger associations with GDM (SUA: OR 1.885, *p* = 0.001; SUA/SCr: OR 1.758, *p* = 0.002) (**Table 3**).

**Table 2:** (A, B) Multivariate logistic regression – GDM incidence.

	B	Standard Error	OR	95% CI	p-Value
Uric Acid	0.004	0.001	1.004	[1.001, 1.006]	**0.003**
Age	0.128	0.026	1.136	[1.080, 1.195]	**0.001**
BMI	0.034	0.037	1.035	[0.963, 1.112]	0.349
Blood Glucose	0.504	0.183	1.655	[1.155, 2.372]	**0.006**
	B	Standard Error	OR	95% CI	p-Value
Uric Acid/Creatinine	0.206	0.071	1.226	[1.070, 1.412]	**0.003**
Age	0.128	0.026	1.136	[1.080, 1.195]	**0.001**
BMI	0.040	0.036	1.041	[0.970, 1.117]	0.265
Blood Glucose	0.508	0.185	1.662	[1.156, 2.391]	**0.006**

Multivariate logistic regression. Bold p-values indicate that the association is statistical significant (p<r0.05).

B, Regression coefficient; BMI, body mass index; CI, Confidence Interval for OR; OR, odds ratio.

**Table 3:** (A, B) Multivariate logistic regression using binary ROC cut-offs – GDM incidence.

	B	Standard Error	OR	95% CI	p-Value
Uric Acid (binary)	0.634	0.198	1.885	[1.279, 2.780]	**0.001**
Age	0.126	0.026	1.135	[1.079, 1.194]	**<0.001**
BMI	0.038	0.036	1.039	[0.968, 1.115]	0.287
Blood Glucose	0.507	0.185	1.660	[1.155, 2.386]	**0.006**
	B	Standard Error	OR	95% CI	p-Value
Uric Acid/Creatinine (binary)	0.564	0.180	1.758	[1.234, 2.503]	**0.002**
Age	0.123	0.026	1.130	[1.075, 1.189]	**<0.001**
BMI	0.042	0.036	1.043	[0.972, 1.119]	0.245
Blood Glucose	0.507	0.187	1.660	[1.151, 2.394]	**0.007**

Multivariate logistic regression. The binary cut-off value (5.02) for the uric acid/creatinine ration was calculated using ROC-analysis and the Youden-Index. Bold p-values indicate that the association is statistical significant (p<0.05).

B, Regression coefficient; BMI, body mass index; CI, Confidence Interval for OR; OR, odds ratio.

**Figures 1a–c** display interaction plots between SUA/SCr and other pre-pregnancy risk factors for GDM. High pre-pregnancy SUA/SCr levels combined with advanced maternal age, higher BMI, and elevated blood glucose were associated with increased GDM incidence in this cohort of women undergoing IVF/ICSI. Subgroup analysis (**Figure 4**) revealed that women with lower pre-pregnancy blood glucose, and higher systolic and diastolic blood pressure particularly benefitted from lower SUA/SCr ratios in terms of GDM risk reduction.

## 4 Discussion

In this study, we evaluated the association between SUA, measured before conception and any reproductive treatments, and GDM incidence, specifically in women undergoing IVF/ICSI. Both pre-pregnancy SUA and SUA/SCr ratio were significantly associated with the occurrence of GDM during pregnancy, independent of known confounding factors.

GDM remains one of the most common pregnancy complications globally, with reported prevalences ranging from 9.5% in Africa to 26.6% in Southeast Asia (15). At the same time, the percentage of affected women has been increasing steadily in recent decades due to demographic changes and increasingly sedentary lifestyles. The overall national prevalence in China is estimated at 14.8% according to Gao et al. (16), though there are significant regional variations. In our IVF/ICSI cohort, 15.5% of the women were diagnosed with GDM during pregnancy, consistent with studies indicating that women undergoing ART are at higher risk (17, 18). This elevated risk is related to the high-risk profile metabolic and hormonal profiles common among women with infertility, including conditions such as polycystic ovarian syndrome (PCOS), a known risk factor for GDM. Additionally, the use of IVF/ICSI has increased with technological advances and increasing maternal age. In China, around 1% of pregnancies were conceived through ART in 2011 (19), whilst in 2023, around 3% of live births had been conceived through ART (20). Given the heightened risk for pregnancy complications in ART patients, early prevention and detection are especially important.

Moreover, in the last decades, there has been a global increase in uric acid levels and the prevalence of hyperuricemia. In our cohort, 173 (16.8%) women had uric acid levels above the general accepted threshold of <357 µmol/L (<6 mg/dL) (21). *The China Nutrition and Health Surveillance* (2015–2017) estimated the overall national prevalence of hyperuricemia to be at 15.1%, whereby males had significantly higher SUA levels than females (21.2% vs. 8.5%) (22). In comparison to 8.5%, the rate of hyperuricemia is notably higher in our cohort, possibly due to infertility-related metabolic factors. Importantly, more recent studies have emphasized that there is currently no universal threshold for uric acid during pregnancy and have suggested much lower cut-off values such as 226 µmol/L (23) and 240 µmol/L (24), though these are not specific to ART populations. In our study, the optimal cut-off values identified through ROC analysis (270.05 µmol/L for SUA and 5.02 for the SUA/SCr ratio) showed a moderate predictive value for GDM. These thresholds may help identify women at increased metabolic risk in early pregnancy or even before. However, given that they were derived from a specific study population, their generalizability to other populations (e.g., with different ethnic backgrounds, BMI ranges, or comorbidities) remains uncertain. Further prospective studies and external validation in diverse cohorts are necessary before clinical application, since our study was focused on Han Chinese women undergoing ART.

While hyperuricemia is traditionally associated with gout, it is also linked to cardiovascular and metabolic diseases (25). We found a statistically significant association between pre-pregnancy SUA levels and GDM, even after adjustment for confounding factors. Previous studies have reported a similar association, but SUA was typically measured during pregnancy, mostly in the first trimester (i.e., prior to the GDM screening) (24, 26, 27). Only one study assessed pre-pregnancy SUA (within six months) and found a significant association between SUA and GDM occurrence (28). A meta-analysis including 11 studies that measured SUA at different points in pregnancy, found that the association was strongest in the first trimester (OR 3.978 [95%CI: 2.177-7.268]) (12). As SUA levels typically decline in early pregnancy due to increased glomerular filtration (24), elevated SUA levels in the first trimester may indicate a maladaptation to pregnancy. Pre-conceptional uric acid levels may be less volatile and could correspond to an already high-risk metabolic profile in line with the CKM syndrome. It is a unique possibility to measure SUA before any reproductive measures in women undergoing IVF/ICSI, since they all undergo baseline testing.

Furthermore, most published studies focused on natural conceptions or did not disclose ART usage. In those studies where data concerning ART usage was included, the proportion was very small (<5%). Although we did not find any studies that focused solely on women undergoing IVF/ICSI, there were two large-scale studies, which included data on whether ART had been used. A study conducted by Zhao et al. with 84,522 patients included 1.3% who had undergone ART (26), whereby these women had a disproportionally higher incidence of GDM (23.6%) compared to women who conceived naturally (13.8%). However, the relationship between SUA and GDM was not evaluated specifically for these patients. In another study conducted in Shanghai, 4.9% of 23,843 study participants had conceived through IVF. Interestingly, this study suggests that especially women with a low or medium risk for GDM could benefit from uric acid measurements to uncover a hidden GDM risk early on (29). Another study only found an association between uric acid and GDM in women younger than 35 years (30). On the other hand, Duo et al. reported that the association between uric acid and GDM was especially strong in older (≥35J) and heavier (BMI ≥ 24 before pregnancy) women in their cohort, suggesting that the metabolism of these women might be influenced more strongly by an elevated uric acid level (24). In our subgroup analysis, lower SUA levels were particularly beneficial for women with a low-risk profile (low blood glucose and low systolic blood pressure) benefitted from a lower uric acid level in terms of their GDM risk. Evidently, more detailed analyses of subgroups would be recommended to clarify these associations.

We also found that the SUA/SCr ratio had an even stronger association with GDM than SUA alone. Since renal function influences SUA levels, adjusting for creatinine offers a more accurate reflection of endogenous uric acid (31). Several studies have shown that the SUA/SCr ratio is associated with metabolic syndrome (31, 32) and a large scale prospective study of 33,030 women found that specifically the SUA/SCr ratio was strongly associated with GDM and pregnancy-induced hypertension (11). Future studies in this field should consider incorporating this parameter into GDM risk assessments.

Whether SUA plays a causal role in GDM remains uncertain. It is known that hyperuricemia is associated with other components and risk factors of the CKM syndrome, such as obesity and an unhealthy diet, which together increase the risk of cardiovascular and metabolic diseases such as diabetes (5). A study using Mendelian randomization analysis had found significantly higher uric acid levels in early pregnancy in women who developed GDM; however, they did not find any genetic evidence for a causal relationship (6). On the other hand, there have also been several proposed mechanisms by which elevated SUA levels could directly cause insulin resistance and thus diabetes. A study by Johnson et al. observed that metabolic syndrome and liver steatosis in rats could be prevented by inhibiting uric acid synthesis (33), and in another study, only fructose-fed rats developed metabolic syndrome, while dextrose-fed rats did not (34). High fructose intake can increase SUA levels because fructose can also be metabolized to uric acid in several steps, unlike the metabolism of other carbohydrates (33, 34). The accumulation of uric acid can have several negative impacts. For one, it can reduce the availability of nitric oxide (NO), which insulin requires for glucose uptake, thus resulting in decreased glucose uptake. Consequently, an elevated level of glucose in the bloodstream and insulin resistance could lead to the development of GDM. Additionally, hyperuricemia has also been linked to endothelial dysfunction through the inhibition of acetylcholine-mediated vasodilation, causing vasoconstriction, also in kidneys, which can lead to the further accumulation of uric acid, as its excretion is reduced. Furthermore, uric acid can cause inflammation and oxidative stress in adipocytes through mechanisms such as an inhibition of adiponectin synthesis (35), which can also regulate blood glucose levels through the inhibition of gluconeogenesis and stimulation of the lipid metabolism, and increase insulin sensitivity (36). In a mouse model, one study showed that uric acid can also influence insulin signaling directly, which can lead to insulin resistance (37). The mechanisms by which uric acid could influence the development of GDM are very complex and remain to be further studied.

Strengths of our study include a relatively large, homogenous cohort of IVF/ICSI patients, a population at increased risk of developing GDM. The study participants had a similar ethnic background and cohort characteristics with relatively small IQRs. SUA was measured preconceptionally, and the SUA/SCr ratio was included to adjust for renal function, which had an even stronger association with GDM. However, the focus on a highly specific population can also be considered a limitation because it restricts the generalizability of our findings. Further studies in other populations are necessary to confirm and extend these results. Another important limitation is that the data were analyzed retrospectively, meaning that several factors, such as certain cardiovascular and metabolic risk factors, including smoking status, dietary habits, and family history of cardiovascular and/or metabolic diseases, were not available, as they were not recorded in the primary study. Furthermore, although a correlation between SUA and the SUA/SCr ratio with GDM was observed, a causal relationship cannot be established based on our data.

Considering the results of our study, SUA, which is simple and fast to measure, could be evaluated to assess individual GDM risk even before conception, together with other known risk factors for GDM. This could enable early preventative measures, such as lifestyle changes as well as an earlier GDM screening for high-risk patients. Future studies could implement the development of a prediction model, which Niu et al. have shown with age, BMI, HbA1c in early pregnancy, lipid profile, and uric acid (38), or a score to easily quantify the risk for GDM before pregnancy. Through early preventative measures, the incidence as well as short- and long-term consequences of GDM could be reduced. We also suggest further analysis of SUA or SUA/SCr levels throughout pregnancy after ART to examine their correlation with GDM, as well as their association with GDM severity.

Although there is emerging interest in uric acid as a potential indicator for early GDM detection, there is very limited evidence regarding interventions targeting SUA to prevent GDM. A possibility that remains to be further studied is whether uric acid-lowering therapy before or during pregnancy – for women with significantly elevated levels – could reduce the incidence of GDM. However, the safety of such treatments (e.g., allopurinol) during pregnancy is not comprehensively known (39). Outside the context of pregnancy, lowering uric acid was shown to modify insulin resistance in preclinical or smaller human studies (35, 40, 41). A meta-analysis by Chen et al. has shown that allopurinol significantly decreased fasting blood glucose, but only in patients without diabetes and only with higher doses of the medication (41). High-quality studies are needed to assess whether these findings translate to pregnant women.

In summary, in our cohort of 1027 women undergoing IVF/ICSI, both pre-pregnancy SUA and SUA/SCr were independently associated with GDM incidence. Given the elevated risk of GDM in this specific population, it is particularly important to assess risk factors to optimize prevention strategies.

## Data Availability

The original contributions presented in the study are included in the article. Further inquiries can be directed to the corresponding author.
